# Nebulized Potassium-Conjugated Linoleic Acid (K-CLA) Therapy in a Patient With Chronic Obstructive Pulmonary Disease and Centrilobular Emphysema: A Case Report

**DOI:** 10.7759/cureus.113447

**Published:** 2026-07-27

**Authors:** Sven T Jonsson, William Beckworth

**Affiliations:** 1 Family Medicine, AdventHealth Hendersonville, Hendersonville, USA; 2 Physical Medicine and Rehabilitation, AdventHealth Hendersonville, Hendersonville, USA

**Keywords:** borg scale, centrilobular emphysema, chronic cough, copd assessment test (cat) score, copd: chronic obstructive pulmonary disease, exertional dyspnea, mmrc dyspnea scale, productive cough, surfactant

## Abstract

Chronic obstructive pulmonary disease (COPD) is a progressive respiratory disorder characterized by persistent airflow limitation that commonly causes dyspnea and reduced exercise capacity. Emphysema, a major component of COPD, involves destruction of alveolar tissue and loss of elastic recoil, resulting in impaired gas exchange and air trapping. We report a 60-year-old female with COPD and centrilobular emphysema who underwent nebulized potassium-conjugated linoleic acid (K-CLA) therapy for persistent dyspnea and exercise intolerance despite standard inhaled therapy. Following two months of treatment, the COPD Assessment Test (CAT) score improved from 23 to 7; the modified Medical Research Council (mMRC) dyspnea grade improved from 2 to 0; Borg Dyspnea and Fatigue scores improved substantially following exertion; and the 6-Minute Walk Test (6MWT) distance increased from 1,345 ft to 1,410 ft. Pulmonary function testing demonstrated mild improvement in FEV1 and the FEV1/FVC ratio without bronchodilator responsiveness. Residual volume (RV) increased, although spirometry-derived estimates of RV should be interpreted cautiously and require confirmation with more definitive lung-volume measurements. No adverse respiratory effects or oxygen desaturation occurred during therapy. These findings suggest potential symptomatic benefits and support further controlled investigation of nebulized K-CLA therapy in obstructive airway disease.

## Introduction

Chronic obstructive pulmonary disease (COPD), including emphysema, is characterized by chronic airflow limitation, dyspnea, exercise intolerance, and progressive deterioration in quality of life. Despite the availability of multiple pharmacologic and non-pharmacologic therapies, many patients continue to experience persistent respiratory symptoms and impaired exercise capacity [1,2].

Potassium-conjugated linoleic acid (K-CLA) is a water-soluble salt form of conjugated linoleic acid (CLA), a family of positional and geometric isomers of linoleic acid (LA), an essential fatty acid. The potential therapeutic effects of K-CLA remain investigational, and the current evidence supporting its proposed mechanisms is derived predominantly from preclinical and in vitro studies. Experimental studies have demonstrated that K-CLA and related CLA derivatives have anti-inflammatory, antimicrobial, antiviral, and surfactant-related properties [3-5]. In vitro investigations have demonstrated substantial reductions in SARS-CoV-2 viral activity at low concentrations (4.4-50.2 mM) of K-CLA [6].

Pulmonary surfactant plays an essential role in maintaining alveolar stability by reducing surface tension. In addition, surfactant can penetrate airway secretions, reducing mucus viscosity and facilitating mucociliary clearance. Beyond these biophysical effects, surfactant processes immunomodulatory properties that contribute to innate pulmonary defense [7].

COPD is characterized by excessive mucus production, which contributes to airway obstruction through the formation of mucus plugs within the airways. These mucus plugs impair airflow, reduce gas exchange, and worsen respiratory symptoms, making restoration of effective mucus clearance an important therapeutic target in COPD [8]. CLA possesses amphiphilic physicochemical properties and can self-assemble into micelles, demonstrating behavior characteristic of surfactants [9]. It is thus plausible that K-CLA may lower interfacial tension within airway secretions, thereby promoting mucus dispersion and mucociliary clearance. Although this mechanism remains hypothetical and has not been demonstrated clinically, it provides a biologically plausible rationale for further investigation.

A recent Cureus case series evaluating the safety of nebulized K-CLA in patients with COVID-19 respiratory tract infections reported that the therapy was well tolerated, with only three discontinuations among 57 treated subjects due to transient adverse effects. The same report described anecdotal improvements in dyspnea and oxygenation, with 18 of 25 hypoxic patients demonstrating improved home pulse oximetry readings following treatment. These observations were exploratory and were not designed to establish clinical efficacy and should be interpreted cautiously. Based on these surfactant-related physicochemical properties of K-CLA, the authors hypothesize that nebulized K-CLA might provide benefit in other mucopurulent pulmonary conditions, emphasizing the need for prospective clinical trials [10].

This case report describes changes in respiratory symptoms, exercise tolerance, laboratory parameters, and pulmonary function measures following two months of nebulized K-CLA therapy in a patient with COPD and centrilobular emphysema. Given the uncontrolled nature of single-patient observation, these observations should be considered hypothesis-generating.

Verbal informed consent was obtained from the patient prior to treatment. Institutional review board approval was not required for this single-patient case report in accordance with institutional policy.​​​​​​

## Case presentation

A 60-year-old woman with COPD and computed tomography (CT)-confirmed centrilobular emphysema underwent baseline evaluation prior to initiation of nebulized K-CLA therapy. She was a former smoker with an 84-pack-year history and had stopped smoking in December 2024. She reported exertional dyspnea, particularly while climbing stairs. Her comorbid history included prediabetes, hypercholesterolemia, obstructive sleep apnea (OSA), elevated coronary artery calcium score, essential hypertension, generalized anxiety disorder, and obesity.

Maintenance COPD therapy consisted of the budesonide/formoterol 160/4.5 mcg inhaler twice daily and an albuterol inhaler as needed. Other medications included irbesartan/hydrochlorothiazide, atorvastatin, nortriptyline, and venlafaxine extended release; OSA was treated with continuous positive airway pressure (CPAP) and 3 L/min nocturnal oxygen.

**Figure 1:**
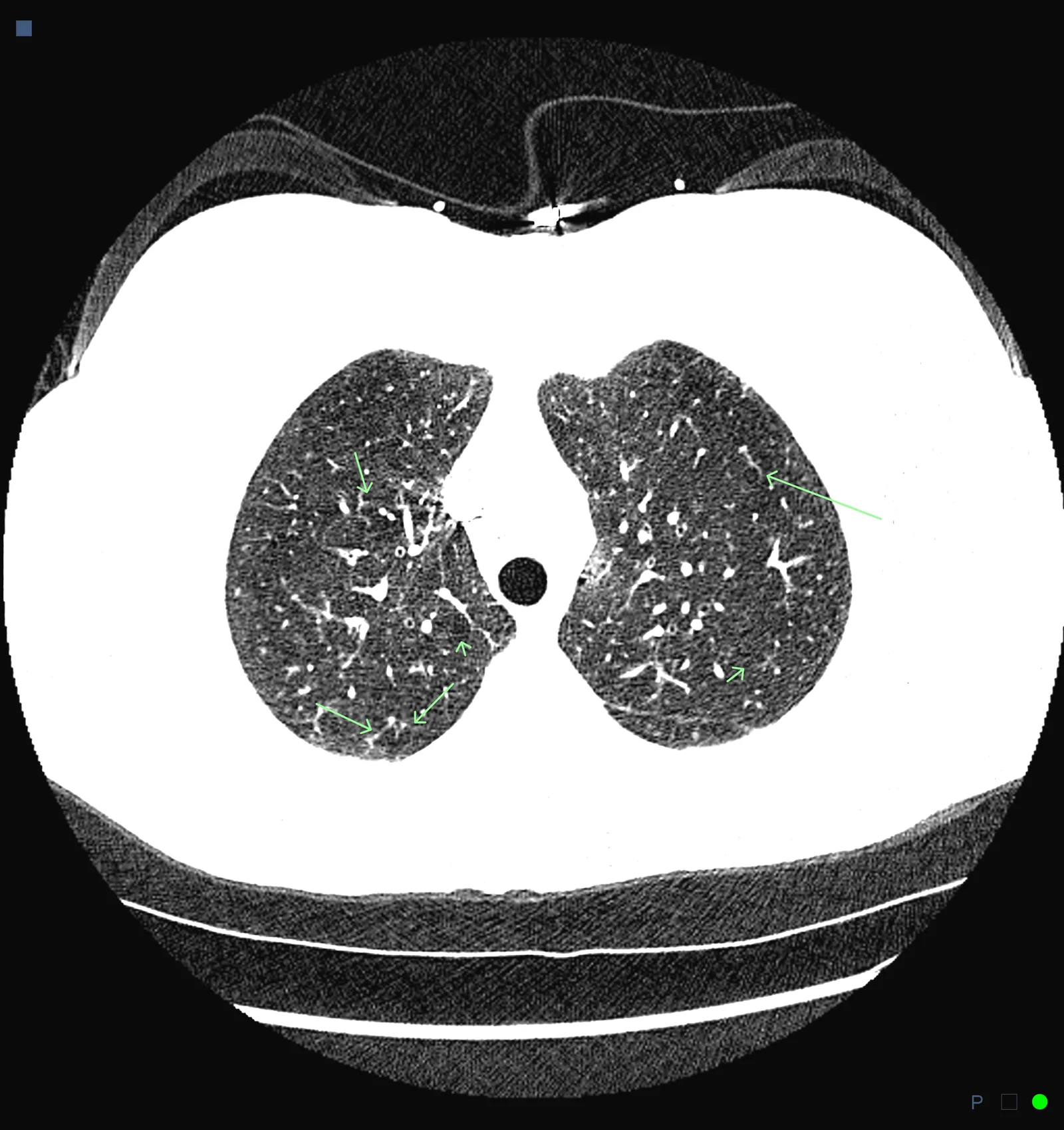
Axial CT pulmonary angiogram demonstrating bilateral centrilobular emphysema Mild centrilobular emphysema is present, affecting the proximal respiratory bronchioles, characterized by round, lucent foci (arrows) involving the central pulmonary lobular unit with upper-lobe (apical) predominance. Mild centrilobular emphysema involves 0.5%-5% of the lung zone.

Baseline CT pulmonary angiography demonstrated mild bilateral centrilobular emphysema with upper-lobe predominance (Figure 1).

Baseline symptoms and functional assessment provided a CAT (COPD Assessment Test) score of 23, an mMRC (modified Medical Research Council) dyspnea grade of 2, and a 6-minute walk distance of 1,345 ft, with post-walk Borg dyspnea and fatigue scores of 7 (Tables 1, 2). Baseline pulmonary function testing showed persistent obstruction, with FEV1 73% predicted, FEV1/FVC ratio 0.60, RV (residual volume) 142% predicted, and DLCO (diffusing capacity of the lung for carbon monoxide) 72% predicted. She had three outpatient exacerbations of her COPD in the previous 12 months. She was classified as GOLD 2, Group E (Table 3) [1].

**Table 1:** Timeline of nebulized K-CLA therapy and clinical outcomes Abbreviations: CAT: COPD Assessment Test; mMRC: Modified Medical Research Council; 6MWT: 6-Minute Walk Test

Date	Test/Treatment/Assessment	Results/Treatment
January–March 2026	CT Angio Chest with IV Contrast LDCT chest	Mild centrilobular emphysema, no pulmonary nodules
March 2026	Complete Pulmonary Function Test	FEV1 73% predicted; FEV1/FVC ratio 0.60; RV 142% predicted
March 2026	Baseline Assessment	CAT score 23, mMRC 2; 6MWT 1345 ft; Borg Score following 6MWT: Dyspnea 7, Fatigue 7
	Treatment	5 mL K-CLA of 11 mM K-CLA diluted with 15 mL distilled water twice daily using a breath-actuated-nebulizer (BAN) initiated following baseline testing Mucinex DM discontinued
April 2026	Dose escalation	10 mL K-CLA of 11 mM diluted with 10 mL distilled water twice daily using BAN
May 2026	Complete Pulmonary Function Test	FEV1 79% predicted; FEV1/FVC ratio 0.62; RV 160% predicted.
May 2026	Follow-up Assessment	CAT score: 7; mMRC: 0; 6MWT: 1410 ft; Borg score following 6MWT: Dyspnea 1, Fatigue 0.5

**Table 2:** Comparison of clinical and functional outcomes before and after nebulized K-CLA therapy Abbreviations: 6MWT: 6-Minute Walk Test; K-CLA: Potassium-Conjugated Linoleic Acid; CAT: COPD Assessment Test; mMRC: Modified Medical Research Council

Characteristic	Pre K-CLA (March 2026)	Post K-CLA (May 2026)
CAT score	23	7
mMRC dyspnea grade	2	0
6-minute walk distance (ft)	1345	1410
Pulse-oximetry at rest (%)	94	95
Pulse-oximetry after 6MWT (%)	97	97
Borg fatigue scale at rest	1	0
Borg dyspnea scale at rest	0.5	0
Borg fatigue scale after 6MWT	7	0.5
Borg dyspnea scale after 6MWT	7	1

**Table 3:** Comparison of pulmonary function test results before and after nebulized K-CLA therapy Abbreviations: FEV1: Forced Expiratory Volume in One Second; FVC: Forced Vital Capacity; FEV1/FVC: Ratio of Forced Expiratory Volume in One Second to Forced Vital Capacity; TLC: Total Lung Capacity; RV: Residual Volume; DLCO: Diffusing Capacity of the Lung for Carbon Monoxide; K-CLA: Potassium-Conjugated Linoleic Acid

Parameter	Pre-Nebulized CLA	Post-Nebulized CLA	Change
FEV1/FVC ratio	0.60	0.62	Mild improvement
FEV1 (% predicted)	73%	79%	Improved
FVC (% predicted)	97%	103%	Improved
Bronchodilator response	None	None	Unchanged
TLC (% predicted)	116%	119%	Stable
RV (% predicted)	142%	160%	Increased
DLCO (% predicted)	72%	71%	Stable
Flow-volume loop	Obstructive defect	Obstructive defect	Persistent obstructive defect

Her prescribed medication regimen was unchanged during the study period. Prior to initiation of nebulized K-CLA therapy, she had been taking over-the-counter dextromethorphan/guaifenesin (Mucinex DM) daily for chronic cough with perceived benefit. Mucinex DM was discontinued when K-CLA therapy was initiated, and no additional antitussive medications were used during the treatment period.

Nebulized K-CLA therapy was started in March 2026. An 11-mM K-CLA formulation (Quorum Sense Now, Ceela Naturals, Louisville, KY, USA) was administered using a reusable breath-actuated nebulizer (BAN). During the first three weeks, 5 mL of the 11 mM K-CLA solution was diluted with 15 mL of distilled water and was nebulized twice daily. This delivered approximately 1.75 mg of K-CLA per treatment. Beginning in week 4, the dose was increased to 10 mL of the 11 mM K-CLA solution diluted with 10 mL of distilled water and nebulized twice daily, delivering approximately 3.5 mg K-CLA per treatment through the remainder of the study period (Table 1).

Clinical outcomes improved, with substantial reductions in symptom burden and improved exercise tolerance following treatment. CAT score [11] decreased from 23 to 7, and the mMRC [12] dyspnea grade improved from 2 to 0. Six-minute walk distance [13] increased from 1,345 ft to 1,410 ft. Post-walk Borg dyspnea and fatigue [14] scores also improved, decreasing from 7 to 1 and from 7 to 0.5, respectively (Table 2).

Pulmonary function testing demonstrated modest improvement in airflow limitation, with FEV1 increasing from 73% to 79% predicted and FVC from 97 to 103% predicted. The FEV1/FVC remained obstructed, with minimal improvement from 0.60 up to 0.62, while DLCO remained stable. RV increased from 142% to 160% predicted despite symptomatic and spirometry improvement. This finding suggests persistent or worsening of air trapping (Table 3).

All eight CAT symptom domains, each scored from 0 (no impairment) to 5 (severe impairment), showed improvement (Table 4). The CAT assesses cough, sputum production, chest tightness, breathlessness when walking uphill or climbing stairs, activity limitation at home, confidence leaving home, sleep quality, and energy level. The greatest reductions were observed in activity limitation at home (4-point decrease), breathlessness when walking uphill or climbing stairs (3-point decrease), and energy level (3-point decrease). Improvements were also noted in cough, sputum production, chest tightness, confidence leaving home, and sleep quality. The composite CAT score decreased from 23 to 7, indicating a marked reduction in symptom burden and a clinically meaningful improvement in health status, exceeding the established minimum clinically significant difference of 2 points.

**Table 4:** Comparison of CAT score symptom domain before and after nebulized K-CLA therapy Abbreviations: K-CLA: Potassium-Conjugated Linoleic Acid; CAT: COPD Assessment Test

Symptom Domain	CAT Score Change	Clinical Implication
Cough	3 to 2	Mild reduction in cough frequency
Sputum production	3 to 2	Mild reduction in chest phlegm
Chest tightness	2 to 0	Complete resolution of chest tightness
Breathlessness climbing stairs or hills	5 to 2	Marked improvement in exertional dyspnea
Limitation of activities at home	4 to 0	Complete resolution of activity limitation within the home
Confidence leaving home	2 to 0	Increased confidence and elimination of disease-related restrictions on leaving home
Sleep quality	1 to 0	Resolution of sleep disturbance attributable to respiratory symptoms
Energy level	4 to 1	Marked improvement in fatigue and overall energy
Overall symptom burden	23 to 7	Substantial reduction in symptoms, representing a 16-point improvement in CAT score

In addition to improvement in spirometry and CAT scores, the patient reported a marked improvement in functional capacity and exercise tolerance. One week before the final follow-up evaluation, she attended a concert that required parking more than 2 miles from the venue. She was able to walk to the concert without stopping to rest, remain active throughout the event, and then return to the vehicle without requiring breaks. She stated that she had never previously been able to accomplish this level of exertion without interruption. Her son, who accompanied her to the event, remarked that he was surprised by how well she tolerated the walking and activities associated with the concert. These subjective improvements were consistent with the reductions in dyspnea and activity limitation reflected by the CAT scores, particularly the improvement in exertional dyspnea and limitation in daily activities. These anecdotal observations represent patient-reported functional improvement and are presented as supportive clinical context rather than objective evidence of treatment efficacy.

Laboratory testing showed no clinically significant safety signal during treatment. The white cell count decreased from 13.05 to 11.1 ×10³/µL, CRP remained stable at 1.29 to 1.28 mg/dL, creatinine remained unchanged at 1.07 mg/dL, and liver enzymes remained within the normal range. Lipid parameters improved, including total cholesterol from 175 to 142 mg/dL, triglycerides from 172 to 148 mg/dL, and LDL cholesterol from 99 to 70 mg/dL (Table 5).

**Table 5:** Comparison of the laboratory findings before and after nebulized K-CLA therapy Abbreviations: K-CLA: Potassium-Conjugated Linoleic Acid; CRP: C-Reactive Protein; AST: Aspartate Aminotransferase; ALT: Alanine Aminotransferase; HDL: High-Density Lipoprotein; LDL: Low-Density Lipoprotein

Laboratory Parameter	Pre K-CLA	Post K-CLA
White cell count (×10³/µL)	13.05	11.1
Neutrophils (×10³/µL)	7.78	6.44
Lymphocytes (×10³/µL)	4.0	3.52
Platelets (×10³/µL)	336	339
CRP (mg/dL)	1.29	1.28
Hemoglobin A1c (%)	6.2	6.1
Creatinine (mg/dL)	1.07	1.07
AST (U/L)	23	26
ALT (U/L)	19	21
Glucose (mg/dL)	128	111
Triglycerides (mg/dL)	172	148
Total cholesterol (mg/dL)	175	142
HDL cholesterol (mg/dL)	41	41
LDL cholesterol (mg/dL)	99	70

## Discussion

This case demonstrated substantial symptomatic and functional improvement following nebulized K-CLA therapy in a patient with COPD and centrilobular emphysema. Respiratory symptom burden decreased markedly, with CAT score improving from 23 to 7, exceeding the established minimum clinically important difference (MCID) of 2 points [15]. Similarly, the mMRC dyspnea grade decreased from 2 to 0, surpassing the reported MCID of 1 point [16]. Measures of exertional symptoms also demonstrated marked improvement, with Borg fatigue scores declining from 7 to 0.5 and Borg dyspnea scores decreasing from 7 to 1 following the 6-minute walk test (6MWT). Both changes exceeded the accepted MCID threshold of 1 point [17]. Exercise capacity increased with a 6MWT improvement from 1,345 ft to 1,410 ft. These changes suggest a clinically meaningful improvement in health status and functional capacity.

Despite advances in pharmacological management, COPD remains a leading cause of morbidity and mortality worldwide. Although guideline-directed pharmacotherapy reduces symptoms and exacerbation frequency, no currently available medical therapy reverses established emphysematous destruction or restores lost alveolar structure. Consequently, there remains considerable interest in novel therapeutic approaches that improve mucus clearance, reduce airway inflammation, enhance surfactant function, or slow disease progression. Recent reviews have highlighted the continued need for therapies targeting mechanisms beyond bronchodilation and inhaled corticosteroids [1,2,18].

Despite substantial symptomatic improvement, pulmonary function testing continued to demonstrate fixed airflow obstruction without bronchodilator responsiveness, consistent with COPD. Small improvements were observed in FEV1 (8.3% relative increase), FVC, and the FEV1/FVC ratio, while diffusion capacity remained stable. RV increased from 142% to 160% predicted, suggesting greater air trapping. Because pulmonary function testing is effort-dependent and subject to biological and technical variability, the isolated increase in RV should be interpreted cautiously [19]. In light of the concurrent improvements in symptoms, exercise capacity, and spirometry indices, the increase in RV remains uncertain and warrants validation in future studies.

Previous studies evaluating nebulized K-CLA in patients with COVID-19 respiratory infections reported a favorable safety profile and suggested potential clinical benefits, particularly with respect to dyspnea and oxygenation. The proposed mechanism was related, at least in part, to the mucolytic properties of K-CLA, which may facilitate the mobilization and clearance of retained airway secretions. In addition, emerging evidence has highlighted a potential role for surfactant-based therapies in the management of viral respiratory illnesses and inflammatory airway diseases [20].

Given the reported in vitro antiviral activity of K-CLA, it is conceivable that nebulized K-CLA could have a role in reducing the risk of respiratory infections or COPD exacerbations. However, this hypothesis cannot be evaluated from a single-patient case report. Furthermore, the observation was limited to two months, precluding any meaningful assessment of exacerbation prevention. Any potential effect on respiratory infections or COPD exacerbations remains speculative and requires validation in prospective controlled clinical studies with longer follow-up and larger patient populations.

No treatment discontinuations, emergency department visits, COPD exacerbations, bronchospasm, or oxygen desaturation events occurred during the treatment period. The primary safety outcome was intolerance to nebulized K-CLA, while secondary safety outcomes included adverse respiratory symptoms and worsening pulse oximetry. No adverse events meeting these criteria were observed.

This report describes the experience of a single patient and therefore cannot establish causality or be generalized to broader patient populations. The absence of a control group limits the ability to distinguish treatment effects from placebo responses or the natural variability of the disease. Improvements in spirometry measures were modest relative to the magnitude of symptomatic improvement. Although discontinuation of daily Mucinex DM at treatment initiation represents a potential confounding factor when interpreting changes in cough and sputum production, the patient reported a transient worsening of cough immediately after discontinuation before subsequent sustained improvement during nebulized K-CLA therapy. This temporal pattern argues against Mucinex DM withdrawal as the primary explanation for the observed clinical improvement, although its potential contribution cannot be completely excluded. Additionally, observed changes may have been influenced by variability in patient effort, increased familiarity with exercise testing, behavioral modifications, or normal fluctuations in COPD symptoms over time. Finally, the short duration of follow-up precludes assessment of long-term safety, durability of response, and effects on COPD exacerbation risk.

## Conclusions

This case demonstrated substantial improvements in respiratory symptoms, exercise tolerance, and selected functional outcomes following nebulized K-CLA therapy in a patient with COPD and centrilobular emphysema, with no apparent short-term safety concerns. Given the uncontrolled nature of a single-patient observation, these results should be considered hypothesis-generating. Further prospective controlled studies are required to determine whether the observed benefits are reproducible and to better characterize the safety and efficacy of nebulized K-CLA in patients with COPD.
